# Regulation of Neuronal Ca_v_3.1 Channels by Cyclin-Dependent Kinase 5 (Cdk5)

**DOI:** 10.1371/journal.pone.0119134

**Published:** 2015-03-11

**Authors:** Aida Calderón-Rivera, Alejandro Sandoval, Ricardo González-Ramírez, Christian González-Billault, Ricardo Felix

**Affiliations:** 1 School of Medicine FES Iztacala, National Autonomous University of Mexico (UNAM), Tlalnepantla, Mexico; 2 Department of Molecular Biology and Histocompatibility, “Dr. Manuel Gea González” General Hospital, Ministry of Health, Mexico City, Mexico; 3 Department of Biology, Faculty of Sciences, University of Chile, Santiago, Chile; 4 Department of Cell Biology, Center for Research and Advanced Studies of the National Polytechnic Institute (Cinvestav-IPN), Mexico City, Mexico; Indiana University School of Medicine, UNITED STATES

## Abstract

Low voltage-activated (LVA) T-type Ca^2+^ channels activate in response to subthreshold membrane depolarizations and therefore represent an important source of Ca^2+^ influx near the resting membrane potential. In neurons, these proteins significantly contribute to control relevant physiological processes including neuronal excitability, pacemaking and post-inhibitory rebound burst firing. Three subtypes of T-type channels (Ca_v_3.1 to Ca_v_3.3) have been identified, and using functional expression of recombinant channels diverse studies have validated the notion that T-type Ca^2+^ channels can be modulated by various endogenous ligands as well as by second messenger pathways. In this context, the present study reveals a previously unrecognized role for cyclin-dependent kinase 5 (Cdk5) in the regulation of native T-type channels in N1E-115 neuroblastoma cells, as well as recombinant Ca_v_3.1channels heterologously expressed in HEK-293 cells. Cdk5 and its co-activators play critical roles in the regulation of neuronal differentiation, cortical lamination, neuronal cell migration and axon outgrowth. Our results show that overexpression of Cdk5 causes a significant increase in whole cell patch clamp currents through T-type channels in N1E-115 cells, while siRNA knockdown of Cdk5 greatly reduced these currents. Consistent with this, overexpression of Cdk5 in HEK-293 cells stably expressing Ca_v_3.1channels upregulates macroscopic currents. Furthermore, using site-directed mutagenesis we identified a major phosphorylation site at serine 2234 within the C-terminal region of the Ca_v_3.1subunit. These results highlight a novel role for Cdk5 in the regulation of T-type Ca^2+^ channels.

## Introduction

The family of voltage-gated Ca^2+^ (Ca_V_) channels are transmembrane proteins that serves as transducers of cell surface membrane potential changes into local intracellular Ca^2+^ transients that initiate a myriad of physiological events. Ca_V_ channels have been traditionally classified into high voltage-activated (HVA) and low voltage-activated (LVA) subtypes [1]. HVA channels activate at relatively depolarized potentials and comprise L-, P/Q-, N-, and R-types. LVA channels, also known as T-type, are critically important for regulating neuronal excitability, pacemaking and post-inhibitory rebound burst firing [2],[3]. Therefore, it should not come as a surprise that T-type channel hyperactivity has been associated to human neurological disorders such as absence epilepsy and neuropathic pain [4],[5],[6],[7].

Three different T-type channels, Ca_V_3.1, Ca_V_3.2 and Ca_V_3.3, have been cloned and expressed from mammals [1],[2]. Using recombinant channels diverse studies have validated the idea that Cav3 channels can be modulated by various endogenous ligands as well as by second messenger pathways. Hence, it has been reported that Ca^2+^/CaM-dependent protein kinase II (CaMKII) differentially regulates the activation of Ca_V_3 channels [8], and that protein kinase A (PKA) and PKC increase Ca_V_3 current density [9],[10],[11]. However, it remains unknown whether other kinases play a role in modulating Ca_V_3 channel function.

Interestingly, it has been shown that the inhibition of the cyclin-dependent kinase 5 (Cdk5) favors neurotransmitter release via enhancement of P/Q-type channel activity [12]. Cdk5 seems to phosphorylate the intracellular loop that connects the second and third repeated domains in the Ca_V_2.1α_1_ pore-forming subunit of the channels, affecting its interaction with SNAP-25 and synaptotagmin [12]. Likewise, recent evidence suggests that the N-type channel, the other major presynaptic Ca^2+^ channel, is also a substrate of Cdk5. In this case, phosphorylation of the Ca_V_2.2α_1_ pore-forming subunit by Cdk5 facilitates neurotransmitter release increasing Ca^2+^ influx by enhancing channel open probability [13].

Cdk5 is a neuron-specific, proline-directed serine/threonine kinase that forms a complex with its activators p35 or p39. Diverse studies have shown that the complex of Cdk5 and its activators has multiple functions in immature neurons including migration, differentiation and synaptogenesis [14],[15]. Although the physiological role of Cdk5 in mature neurons is less clear, it has been suggested that several proteins of the soluble N-ethylmaleimide-sensitive factor attachment protein (SNAP) receptor (SNARE) required for efficient neurotransmitter release may act as physiological substrates of Cdk5. Likewise, it has been documented that proteolytic cleavage of p35 may produce p25, which accumulates in the brain of patients with Alzheimer's disease [13],[16]. Furthermore, increased proteolysis of p35 is linked to abnormal tau phosphorylation and promotes neuronal apoptosis [17].

In the present study we analyzed Ca_V_3.1 channels for potential phosphorylation by Cdk5. We report that Cdk5 can directly phosphorylate Ca_V_3.1 channels at serine 2234 and that this in turn modulates depolarization-dependent Ca^2+^ entry.

## Materials and Methods

### Cell cultures

Mouse neuroblastoma-derived N1E-115 cells (American Type Culture Collection; ATCC Number CRL-2263) were grown in culture using Dulbecco’s modified Eagle’s medium plus 25 mM glucose (DMEM-HG) culture medium supplemented with 10% fetal bovine serum, 2 mM L-glutamine, and penicillin-streptomycin (100 U/mL). Cells were incubated in a humid atmosphere of 5% CO_2_-95% air at 37°C. The incubation medium was changed every 2 days. Cells were harvested once per week by treatment with a trypsin-EDTA solution, and reseeding was carried out at 20% of the original density. Human embryonic kidney (HEK) 293 cells stably expressing the Cav3.1a channel [18],[19] were grown as described elsewhere [20]. In brief, cells were kept in culture in DMEM supplemented with 1 mg/ml G418 (Gibco/BRL Life Technologies), 10% fetal bovine serum, and penicillin-streptomycin (100 U/mL) at 37°C in a 5% CO_2-_95% air humidified atmosphere and sub-cultured by mechanical dispersion every week.

### Electrophysiology

N1E-115 and HEK-293 cells were subjected to the standard whole cell patch-clamp technique using an Axopatch 200B amplifier as described previously [20]. Current signals were filtered at 2 kHz, digitized at 5.71 kHz and analyzed with pClamp software. Data were leak subtracted on line by a P/4 protocol. The bath recording solution contained (in mM) either 5 CaCl_2_ (HEK-293 stable transfected), 2 BaCl_2_ (HEK-293 cells transiently transfected), or 10 BaCl_2_ (N1E-115 cells), 125 TEA-Cl, 10 HEPES and 10 glucose (pH 7.3). The internal solution consisted of (in mM) 110 CsCl, 5 MgCl_2_, 10 EGTA, 10 HEPES, 4 Mg-ATP and 0.1 GTP (pH 7.3). Pipettes with resistance values in the range 2–3 MΩ were used. Linear leak and parasitic capacitance components were subtracted on-line using a P/4 protocol. Membrane capacitance (*C*_m_) was determined as previously described [21] and used to normalize currents.

### Data analysis

Activation of the currents was well described by a Boltzmann relation of the form *G*/*G*_max_ = 1/{1+exp[(*V*-*V*_½_)/*k*]}, where *G* is peak conductance, *G*_max_ is fitted maximal *G*, *V*_½_ is half-activation voltage, and *k* is the slope factor. Steady-state current inactivation curves were fitted with the following equation: *I/I*_max_ = 1/(1+exp(−(*V*_p_−*V*_½inact_)/*k*)), where *I/I*_max_ is the normalized peak current, *V*_p_ is the conditioning prepulse, *V*_½inact_ is the voltage for half-inactivation and *k* is the slope factor. Time constants of activation (τ_act_) and inactivation (τ_inact_) were obtained, using 140 ms test pulses, from single exponential fits to the raising and decaying phases of the currents.

### Cdk5 gene silencing by specific siRNAs

N1E-115 cells were seeded in 35-mm dishes (at ∼80% confluence) and transfected with a 100-pmol mixture of predesigned synthetic small interfering RNAs (siRNAs) that target Cdk5 or with a non-targeting siRNA as negative control (Sigma-Aldrich) using Lipofectamine 2000 transfection reagent (Invitrogen). Twenty-four hours after transfection, cells were harvested to analyze the knockdown effect of siRNAs on endogenous Cdk5 protein levels by Western blot using specific antibodies or re-seeded for electrophysiological recording.

### Western blot

Total protein extracts were obtained using 200 μl of lysis buffer (50 mM Tris-HCl pH 8.0, 150 mM NaCl, 0.5 mM PMSF, 1% Triton X-100, 1X protease inhibitor mix (Roche Applied Science). After centrifugation, supernatants were recovered for protein quantification by the bicinchoninic acid reaction. Aliquots of 50 μg of total protein extracts were boiled in SDS sample buffer (50 mM Tris-HCl pH 6.8, 2% SDS, 10% glycerol, 0.1% 2-mercaptoethanol, 0.001% bromophenol blue), electrophoresed on 10% SDS-polyacrylamide gels and transferred to nitrocellulose membranes (Amersham Pharmacia GE Healthcare). After blocking with non-fat milk (5%) supplemented with 0.2% Tween 20, membranes were incubated 1 h with the primary antibody anti-Cdk5 or anti-p35 (C-8 and C-19, respectively; Santa Cruz Biotechnology) at 1:1000 dilution in TBS-T with 5% non-fat milk (Santa Cruz) washed with TBS-T (10 mM Tris-HCl, 0.15 M NaCl, 0.05% Tween 20), and finally incubated with goat anti-rabbit secondary antibody coupled to horseradish peroxidase for 1 h. Immunoblots were developed by using the ECL Western blotting analysis system (Amersham Pharmacia GE Healthcare).

### Total RNA isolation and RT-PCR

Total RNA was isolated from N1E-115 cells using TRIzol reagent (Invitrogen). Five micrograms of total RNA was reverse transcribed using random hexanucleotides and the M-MLV reverse transcriptase (Invitrogen), according to the manufacturer’s instructions. After inactivation of reverse transcriptase cDNAs were processed immediately for amplification. The PCR reaction was carried out in a total volume of 50 μl containing 5 μl of cDNA solution, 1X PCR buffer (20 mM Tris-HCl pH 8.4, 50 mM KCl), 0.2 mM of each deoxynucleotide triphosphate, 1.5 mM MgCl_2_, 30 pmol of each primer, and 2.5 U of Taq DNA polymerase (Invitrogen) on a PCRsprint thermal cycler (Thermo Scientific). The PCR conditions included an initial denaturation step at 94°C for 5 min followed by a cyclic reaction consisted of denaturation at 94°C for 30 s, annealing at 55°C for 30 s and extension for 1 min at 72°C. PCR products were then subjected to 1% agarose gel electrophoresis and nucleic acid bands were visualized by ethidium bromide staining. The amplified products were purified and sequenced. Primers used in this work are as follows (forward and reverse): 5’-TGCAGAAATACGAGAAACTGGA-3’ and 5’-ACATGTCGATGGACGTGGAGTAC-3’ for CDK5; 5’-AAGAAGAAGAACTCCAAGAA-3’ and 5’-ACATCCCTGCAGAGCATGTA-3’ for p35; 5’-CACTTGTGCACCAGCCACTA-3’ and 5’-AGGTCTCCAAAGAGCTCCAC-3’ for Ca_V_3.1; 5’-TTCTTCAAGGACAGGTGGAA-3’ and 5’-GCCTCCTTGTTGCTCTCCTC-3’ for Ca_V_3.2; 5’-TGTGCCTTCTTCATCATCTT-3’ and 5’-AAGGTGATGAAGATGTCCAG-3’ for Ca_V_3.3; and 5’-AAGATGACCCAGATCATGTT-3’ and 5’-GAGTACTTGCGCTCAGGAGG-3’ for actin.

### Transient transfection of HEK-293 cells

HEK-293 cells (ATCC Number CRL-1573) were maintained in DMEM-HG medium supplemented with 10% horse serum, 1% L-glutamine, 110 mg/L sodium pyruvate and antibiotics, at 37°C in a 5% CO_2-_95% air humidified atmosphere. Gene transfer was performed using Lipofectamine Plus reagent (Invitrogen). Briefly, for a 35-mm Petri dish of HEK293 cells, 1.2 μg of the plasmid cDNA encoding the rat brain T-type Ca^2+^ channel Ca_V_3.1 subunit (GenBank accession number AF027984.1; Perez-Reyes et al., 1998), a generous gift of Dr. JH Lee (Sogang University, Korea), or its S2234A mutant, as well as 1.5 μg Cdk5 and 0.5 μg p35 plasmid constructs (NM_007668.3 and U89527.1, respectively) were premixed with 6 μL of Lipofectamine in 100 μL serum-free medium according to the manufacturer’s instructions.

### Immunofluorescence

HEK-293 cell line stably expressing Ca_V_3.1 channels were grown on poly-L lysine coated slides, fixed with 4% formaldehyde in PBS for 15 min at room temperature (RT) and washed with PBS. Cells were permeabilized with 0.2% Triton X-100 and blocked with 1% gelatin and 10% FBS for 30 min and incubated overnight (at 4°C) with the primary anti Ca_V_3.1 antibody (Millipore at 1:20 dilution in 0.1% Triton X-100, 2% BSA and 5% FBS). Cells were then incubated for 1 h at RT with the secondary antibody (goat anti-rabbit IgG coupled to Cy5; Zymed). After washing slides were mounted with VectaShield (Vector Laboratories) and fluorescence was visualized with a confocal laser scanning microscope (TCS-SP2, Leica) using a 63x oil-immersion objective.

### Plasma membrane protein extraction

HEK-293 cells were transfected with the plasmids codifying for Ca_V_3.1-YFP in the absence (control) or the presence of Cdk5/p35. After 48 h the extraction of membrane proteins was carried out using the Membrane Protein Extraction Kit (Cat # K268-50; BioVision) according to the manufacturer’s instruction. Briefly, cells were grown in p100 culture dishes and collected by centrifugation (700 x g, 5 min), washed with cold PBS, resuspended and homogenized. The homogenate was centrifuged at 700 x g for 10 min. The pellets containing the cell membrane proteins were mixed both with 200 μl of the upper and lower phase solutions and centrifuged at 1000 x g for 5 min. The upper phase was carefully transferred to a new tube, diluted in 5 volumes of water and centrifuged for 30 min. The supernatant was removed, and the pellet containing the membrane proteins was dissolved in 0.5% Triton X-100 in PBS. Proteins were then quantified using the BSA method and separated by SDS-PAGE electrophoresis gel, and transferred to a nitrocellulose membrane. For Ca_V_3.1 channel detection, the membrane was incubated with anti-GFP antibody (1:5000 dilution; Aves Labs) and the loading control protein was detected with an anti-pan-Cadherin antibody (1:50 dilution; Life Technologies). The quantification of the band intensity of the Ca_V_3.1 signal was normalized to Cadherin and expressed as arbitrary units.

### Site-directed mutagenesis

Multiple nucleotide sequences were aligned using the modified Clustal W algorithm of Vector NTI 8 software package (Invitrogen). Phosphorylation site(s) prediction was performed using the GPS 2.0 software (Group-based Prediction System, ver 2.0), a state-of-the-art web-based software for prediction of phosphorylation available at http://gps.biocuckoo.org/. The Ca_V_3.1 cDNA was inserted into the expression plasmid pcDNA3 and expressed under the control of the cytomegalovirus promoter. A point mutation (S2234A) was introduced with ∼40-mer synthetic oligonucleotides using the Quik-Change XL-mutagenesis kit (Stratagene). Mutant channel cDNA was sequenced (PerkinElmer Applied Biosystems).

### Plasmid constructs

pGEX expression constructs encoding rat Ca_V_3.1 C-terminus were generated by amplifying a fragment of ∼300 bp comprising amino acids 2154 to 2254 of the protein using the following primers: forward 5´ GAATTCAAGCGGCTCCCAACCCCGCCT 3´ and reverse 5´ GAATTCGGGGTCCATGTCTGTTGGGT 3´. Amplification was carried out in a PCR Sprint Thermal Cycler (Thermo Scientific) with a preliminary denaturation step at 94°C for 5 min, followed by 30 cycles at 94°C for 45 s, primer annealing at 55°C for 15 s and primer extension at 72°C for 60 s, followed by a 10 min final extension at 72°C. The fragment was then digested with EcoRI and cloned into the EcoRI site of the pGEX-3X vector (pGEX-COOH). Two mutagenic (S2234A) oligonucleotides were designed as follows: forward 5’ CGGCTGCCTCACCCGCCCCAAAGAAAGAC 3’; reverse 5’ GTCTTTCTTTGGGGCGGGTGAGGCAGCCG 3’. One hundred twenty five ng of mutagenic oligonucleotides were used in each synthesis reaction (full-length Ca_V_3.1 or GST-carboxyl terminal) using Quik-Change II XL-mutagenesis kit (Agilent Technologies). Digestion of the amplified products with DpnI preceded transformation of XL10-Gold ultracompetent cells. All cDNAs were sequenced.

### Expression and purification of the Ca_V_3.1 C-terminal constructs

pGEX-COOH vector was transformed into BL21 cells for expression and purification of the GST-COOH proteins. Protein expression was induced with 1.0 mM IPTG after cells reached an absorbance of 0.5 at 595 nm for 5 h. Cells were spun at 4°C for 20 min and resuspended in lysis buffer [10 mM Tris pH 8.0, 150 mM NaCl, 1 mM EDTA pH 8.0, 3% Sarcosyl and 1X protease inhibitor mix (Roche Applied Science)] and sonicated (Vibra-Cell VCX 130) on ice and then centrifuged at >12,000 g for 30 min to pellet the insoluble material. Supernatants were removed and mixed with 1 mL of glutathione agarose (Invitrogen) to purify the proteins. After releasing the carboxyl-terminal from GST with Xa factor, proteins were used in phosphorylation assays.

### *In vitro* phosphorylation

The Ca_V_3.1 C-terminal WT or S2234A constructs were incubated with 100 ng of recombinant human CDK5-p35 complex (Invitrogen) and 2 mM ATP in kinase reaction buffer (20 mM Tris-HCl, pH 7.5, 1 mM MgCl_2_, 1 mM DTT) in a final volume of 20 μL for 1h at 30°C. The reaction was stopped by adding 20 μL of 2X RSB (20 mM Tris HCl pH7.4, 20 mM NaCl, 6 mM MgCl_2_) and 8 μL (6X) SDS sample buffer, and subsequently boiled for 5 min at 90°C. Samples were separated by 15% SDS-PAGE and gel bands revealed by colloidal Coomassie blue staining or treated with Pro-Q Diamond phosphoprotein gel stain (Invitrogen) according the manufacturer's instructions. Gels were fixed with 50% methanol and 10% acetic acid overnight, washed with ddH_2_O and stained with Pro-Q Diamond for 90 min. Gel was destained (20% acetonitrile, 50 mM sodium acetate, pH4) and the phosphoprotein bands detected using a Typhoon instrument (excitation/emission: 532/560 nm; Molecular Dynamics).

## Results

**Fig 1:**
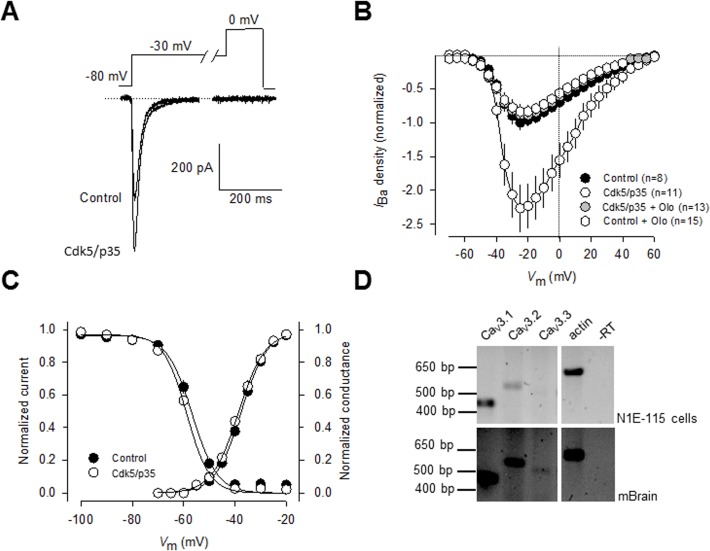
Regulation of native T-type Ca2+ channels by Cdk5. A) Cdk5 transfection significantly increases T-type current amplitude in neuroblastoma N1E-115 cells. Currents were evoked by step depolarizations applied to test potentials of −30 mV from a holding potential (*V*_h_) of −80 mV. Ba^2+^ (10 mM) was used as the charge carrier. B) Normalized current density-voltage relationships in control and transfected cells with Cdk5/p25 in the absence and presence of the Cdk5 inhibitor olomoucine (50 μM), obtained by application of 140-ms voltage steps to various test potentials ranging from −60 to +60 mV from a *V*_h_ of −80 mV. Peak currents were normalized by membrane capacitance (current density, pA∙pF) to eliminate cell size as a variable. C) Cdk5/p35 effect on T-type channels did not alter the voltage dependence of current activation and steady-state inactivation. The normalized curves were fit by a single Boltzmann as described in Methods. D) Ca_V_3 channel expression in N1E-115 cells. Total RNA was isolated and reverse transcribed. Amplified products were resolved by agarose gel electrophoresis. Actin was included as a positive control. RNA samples transcribed in the absence of reverse transcriptase (-RT) were used as negative controls to exclude genomic contamination. Mouse brain (mBrain) was used as a positive control.

To investigate whether T-type (Ca_V_3) channels are substrates of Cdk5, we initially conducted whole-cell patch clamp recordings in the clonal neuroblastoma cell line N1E-115 which express prominent Ca^2+^ currents through Ca_V_3 channels [22],[23]. These transient inward currents (Fig. 1A) were evoked by a step depolarization from a holding potential (*V*_h_) of −80 mV to potentials more positive than −50 mV, and the current amplitude became maximum around −25 mV (Fig. 1B). Likewise, when preceded by a 1 s conditioning pulse to −30 mV to inactivate the T-type component, step depolarizations failed to evoke the long-lasting component of the current previously observed in N1E-115 cells induced to differentiate into mature neurons [22],[23], suggesting that the contribution of L-type channels to the whole-cell current in non-differentiated N1E-115 cells is negligible (Fig. 1A).

The potential effects of Cdk5 on the capacity of T-type Ca_V_ channels to conduct current were then examined using also whole-cell recordings in N1E-115 cells transfected with the Cdk5 plus its activator p35 cDNAs. Using Ba^2+^ as the charge carrier, we found that following transfection with Cdk5/p35, the peak current amplitude (Fig. 1A) and current density were significantly increased (>2-fold) when compared to the control condition (Fig. 1B). The possible role of Cdk5/p35 on T-type current regulation was supported by using the inhibitor olomoucine (50 μM), which fully prevented the effect of the kinase on the macroscopic currents. Inward currents were measured and expressed as peak current density (pA/pF) to account for variations in cell size and the current density-voltage relationships were normalized (Fig. 1B).

Given that changes in current amplitude could result from changes in channel gating, the voltage-dependent properties of the currents were assessed in these cells. Activation of the channels was well described by a Boltzmann relation of the form *G*/*G*_max_ = 1/{1+exp[(*V*-*V*_½_)/*k*]}, where *G* is peak conductance, *G*_max_ is fitted maximal *G*, *V*_½_ is half-activation voltage, and *k* is the slope factor. As shown in Fig. 1C, for control (GFP-transfected) cells, *V*_½_ was −37.5 mV (*n =* 13), which was not significantly different from that of Cdk5/p35-transfected cells (-38.6 mV, *n* = 19; *P*>0.05, Student's t-test). The slope factors for the Boltzmann fits were also similar. Likewise, the voltage-dependent properties of inactivation were determined by applying 1 s conditioning pre-pulses that ranged successively from −80 to −20 mV in 10 mV voltage steps, followed by a 140 ms step depolarization to −30 mV. The relationship of normalized test pulse voltage to peak current amplitude was plotted against its corresponding *V*_h_ and fitted with the Boltzmann equation (Fig. 1C). The voltage dependence of inactivation was similar for currents recorded from control and cells expressing Cdk5 (Fig. 1C). For control cells, *V*_½_ was −56.7 mV (*n* = 13), which was not significantly different from that of Cdk5/p35 transfected cells (-58.7 mV, *n* = 19; Fig. 1C). The slope factors were also similar. These results indicate that Cdk5 augmented the density for currents carried by native T-type channels but had no apparent effect on either the activation or inactivation properties.

We next examined whether one or more of three known members the T-type channel subfamily (Cav3.1, Cav3.2 and Cav3.3) were expressed in N1E-115 cells. To this end, total RNA isolated from N1E-115 cells was isolated and sequence-specific primers were designed to target Ca_V_3 channels in RT-PCR experiments. As previously reported [24] and corroborated here, single Ca_V_3.1 and Ca_V_3.2 products of the expected sizes can be selectively amplified from N1E-115 cells using RT-PCR (Fig. 1D, upper panel). In addition, no significant expression of Ca_V_3.3 mRNA could be detected. In contrast, evidence for the expression of the three Ca_V_3 channel isotypes were found in the total RNA obtained from the mouse brain used as control (Fig. 1D, lower panel).

**Fig 2:**
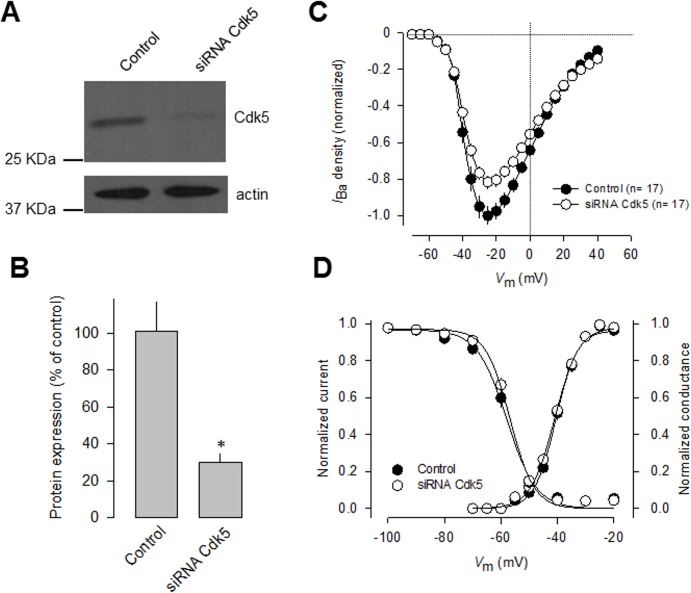
Knockdown of endogenous Cdk-5 decreases T-type Ca2+ channel functional expression. A) N1E-115 cells were transfected with Cdk5 siRNA and analyzed 48 h later by Western blot with specific antibodies, verifying siRNA-mediated reduction of endogenous Cdk5. B) Densitometry quantification of Cdk5 protein in control and siRNA Cdk5-transfected cells (*n* = 3). Asterisk denotes a significant difference at *P* < 0.05. C) Comparison of normalized current density-voltage relationships in control and siRNA transfected cells. Ba^2+^ (10 mM) was used as the charge carrier. D) Cdk5 silencing did not alter the voltage dependence of current activation and steady-state inactivation.

To confirm that Cdk5 manipulation affected T-type current density, we next performed Cdk5 knockdown experiments using small interfering RNAs (siRNAs). We first verified efficient and specific knockdown of the kinase in N1E-115 cells using semi-quantitative Western blot analysis with an anti-Cdk5 antibody (Fig. 2A-B). Loading controls were monitored with an anti-actin antibody. The anti-Cdk5 antibody recognized a 32-kDa protein in N1E-115 cell line, as well as in mouse brain lysates and in HEK-293 cells (Fig. 2A). Our analysis of protein expression levels confirmed a significant decrease (∼70%) in the levels of Cdk5 protein after knockdown.

Interestingly, whole-cell recordings of N1E-115 cells transfected with Cdk5 siRNAs for 48 h showed a significant decrease (∼20%) in T-type current density compared with scrambled siRNA transfected cells. Scaled current density-voltage relationships confirmed that Cdk5 knockdown has an inhibitory effect on T-type current density in N1E-115 cells (Fig. 2C). The discrepancy between the efficiency of the Cdk5 knockdown (protein decrease of ∼70%) and its effect on the T-type channels (current reduction of ∼20%) suggests that there might not be a linear relationship between the silencing of Cdk5 and its effect on T-current density. This is not unexpected given that posttranslational modifications such as phosphorylation work in a non-stoichiometric manner. In this particular case, the amount of Cdk5 remaining after knockdown still could be phosphorylating a substantial proportion of channels. In addition, the Ca_V_3.1 channels conduct current even in the absence of Cdk5-dependent phosphorylation, as could be found for the mutant Ca_V_3.1 channels (see below). Last, voltage-dependent properties of activation and inactivation of the T-type currents were examined in transfected N1E-115 cells, and the results showed that there were no differences in the half-maximal activation (V_½_) and slope factors (*k*) of steady-state activation or inactivation between Cdk5 siRNA-transfected and control cells (Fig. 2D).

**Fig 3:**
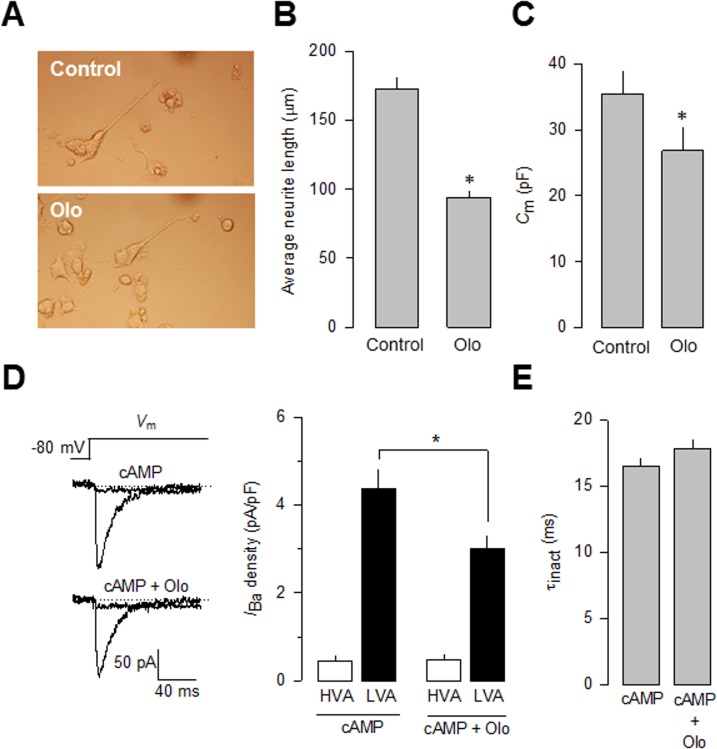
Cdk5 inhibits T-type Ca2+ channel functional expression and affect cAMP-mediated N1E-115 cell differentiation. A) Inhibition of neurite outgrowth by the specific Cdk5 inhibitor olomoucine (Olo) in N1E-115 differentiated with cyclic adenosine monophosphate (cAMP, 2 mM) for 48 h. Phase contrast micrographs of cells grown in the absence or presence of Olo (50 μM). B) Comparison of neurite outgrowth from N1E-115 cells kept in culture in the absence (control) and presence of Olo. Neurite analysis was carried out with ImageJ software (NIH). C) Comparison of the *C*_m_ values in cAMP-differentiated N1E-115 cells kept in culture in the presence or the absence of Olo. D) Representative superimposed trace currents recorded in response to 1 s depolarizing pulses to −30 mV from a *V*_h_ of −80 mV (to evoke LVA channel activity), and to +10 mV at the end of the 1 s LVA current inactivating pulses (to evoke the HVA component of the current) in cAMP-differentiated N1E-115 cells in the presence or the absence of Olo (left panel). Comparison of the percentage of peak current densities through HVA and LVA channels (right panel). Data are given as mean ± S.E.M. E) Comparison of the time constant of current and inactivation (τ_inact_) at −30 mV in cAMP-differentiated N1E-115 cells in the presence or the absence of Olo as in D.

Since T-type channels have been implicated in neuronal differentiation [25], and manipulating Cdk5 could be an opportunity to better understand their role during this period, the role of Cdk5 on cyclic adenosine monophosphate (cAMP)-induced differentiation of the neuroblastoma-derived N1E-115 cells was studied using olomoucine (Olo; 50 μM). The results of these experiments show that the use of this Cdk5 competitive antagonist prevented the effect of cAMP (Fig. 3A). To characterize this effect, neurite incidence and average neurite length were monitored for 48 h in the absence and presence of Olo. Neurite incidence increased at the same rate and kept rising until 48 h. In contrast, neurite length increased to an average of ∼170 μm in the control condition but stopped at 48 h in Olo-treated cells, averaging <100 μm (Fig. 3B). Consistent with this, cell membrane capacitance, determined for these cells as an index of cell size, was smaller in the cells incubated 48 h with Olo (Fig. 3C).

In order to obtain proof for the T-type (Ca_V_3) channel involvement in this process, we next performed whole-cell patch clamp recordings in cAMP-differentiated N1E-115 cells in the absence and the presence of Olo (50 μM). Using Ba^2+^ as the charge carrier, we found that treatment with Olo (48 h) significantly inhibited (∼30%) current density when compared to the control condition (Fig. 3D). As mentioned earlier, it has been reported that L-type (Ca_V_1) channels may be expressed in differentiated NIE-115 cells. Therefore, the possibility exists that the over-expression of Cdk5/p35 might induce their functional expression. To explore this possibility, the effect of Cdk5/p35 on HVA Ca_V_ channel expression was examined using whole cell patch clamp recordings in NIE-115 cAMP-differentiated cells. To this end, a standard protocol was employed. First, a 1 s depolarizing step to −30 mV from a *V*_h_ of −80 mV was applied to activate and inactivate the low voltage-activated (LVA) component of the macroscopic current but not activate HVA channels. Next, a second activating voltage step to 0 mV was applied to activate HVA channels [26]. The results of this analysis suggested that a small amount (∼10%) of HVA (including L-type) inward current could be detected in NIE-115 cells under differentiating conditions (Fig. 3D). Interestingly, this component of the macroscopic current was not affected by Olo treatment. In contrast, the LVA component of the Ca^2+^ current was sensitive to the drug treatment. In the presence of Olo the LVA current density was significantly reduced from ∼−4.5 pA/pF in the control to ∼−3 pA/pF (Fig. 3D).

It should be noted here, that the Cdk5 inhibitor roscovitine (Ro) has also shown to affect Ca_V_3.1 channel activity. Specifically, the drug seems to inhibit these channels in part by stabilizing the closed-inactivated state [27]. In addition, it has been reported that Ro affects Ca_V_2.2 (N-type) current kinetics [28]. However, this effect appears to be specific for Ro since it is not present after Olo treatment [28], which points to distinct mechanisms of action. The differential effects of these two closely related Cdk5 inhibitors may also be true for the Ca_V_3.1 channels, where we found that current inactivation kinetics in control cells and cells treated with Olo did not differ significantly (Fig. 3E).

Although it is likely that the current recorded in N1E-115 cells may be mediated by Ca_V_3.1, Ca_V_3.2, or both channels, in a previous report we showed that the transcription factor Sp1 can regulate Ca_V_3.1 promoter activity and that siRNA-mediated Sp1 silencing significantly decreased the level of Ca_V_3.1 protein and reduced the amplitude of whole-cell T-type currents expressed in the N1E-115 cells [29]. These results indicated that Ca_V_3.1 channels greatly contribute to determine Ca^2+^ macroscopic currents in these cells.

**Fig 4:**
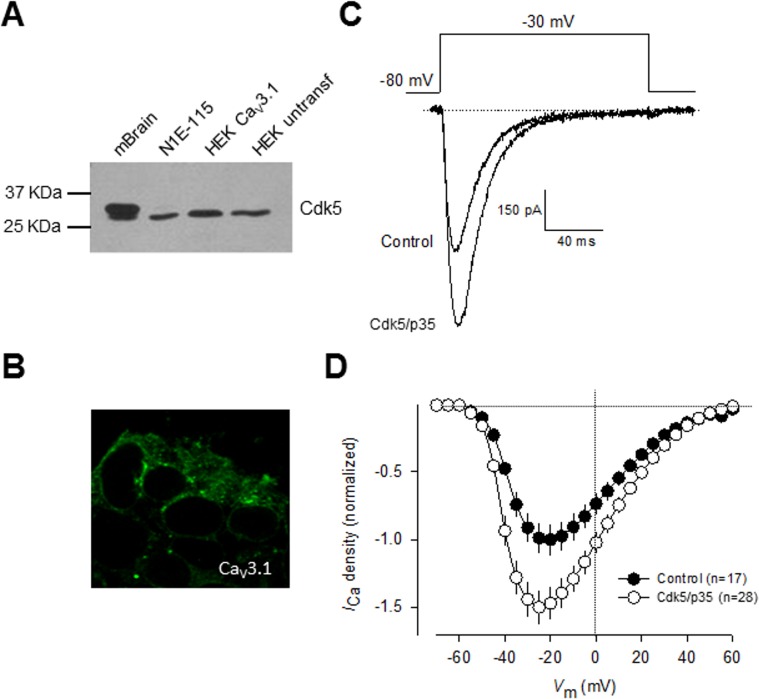
Regulation of heterologously expressed CaV3.1 channels by Cdk5. A) Protein extracted from mouse brain (mBrain), N1E-115 cells, untransfected HEK-293 cells as well as stably expressing Ca_V_3.1 channel cells were analyzed by Western blot using specific antibodies for Cdk5 (upper panel) and its activator p35 (lower panel). B) Immunofluorescence analysis of HEK-293 cells stably expressing Ca_V_3.1 channels. The confocal image illustrates the expression of the channels (green) both in the plasma membrane and the cytosol. Cells were fixed and stained with a polyclonal anti-Ca_V_3.1 antibody. C) Representative macroscopic current traces recorded from HEK-293 cells stably expressing Ca_V_3.1 channels in the control condition and after transfection with plasmids encoding Cdk5 and p35. Currents were elicited by depolarizing steps from a *V*_h_ of −80|mV to −30|mV. Ca^2+^ (10 mM) was used as the charge carrier. D) Comparison of normalized current density-voltage relationships in control and Cdk5/p35 transfected HEK-293 cells.

Consequently, we next investigated the functional significance of Cdk5-mediated phosphorylation on whole-cell currents recorded in HEK-293 cells stably expressing Ca_V_3.1 channels and transiently transfected with the cDNAs encoding for Cdk5 and p35. However, before exploring this point, in an initial series of experiments, cell lysates from mouse brain, N1E-115, and HE-293 cells were subjected to Western blot analysis using anti-Cdk5 and anti-p35 to detect the expression of endogenous Cdk5 and p35 proteins. The results of these experiments revealed bands corroborating the expression of endogenous Cdk5 (Fig. 4A) and p35 in all samples analyzed. However, given that the expression of p35 has not been detected previously in the HEK-293 cell line [30],[31], we decided to verify its expression at the level of mRNA in RT-PCR experiments using the same set of specific oligonucleotides as in Fig. 1A. Unexpectedly, our results showed no specific p35 mRNA amplification in HEK-293 cells. Although there are some possible explanations for the discrepancy between the data obtained by Western blot and RT-PCR, the actual reasons for these conflicting results remain presently unknown. However, given that in all experiments examining the effect of Cdk5 phosphorylation on Ca_V_3.1 channels performed in HEK-293 cells, p35 was co-transfected with the kinase and the channels, whether or not p35 is endogenously expressed in this cell line does not affect the results of this study. Additional studies are needed to unambiguously demonstrate the expression of p35 in the HEK-293 cell line.

We further confirmed the expression of Ca_V_3.1 in the surface of transfected HEK-293 cells by immunofluorescence. As indicated by the green fluorescence signal in Fig. 4B, we found that the Ca_V_3.1 channels show a distribution pattern consistent with predominant plasma membrane expression, though there was also signal associated to cytoplasmic organelles. Likewise, representative current traces elicited near the half-maximal (-30 mV) channel activation are shown in Fig. 4C. Remarkably, a ∼1.5-fold increase in current density was observed in cells transfected with Cdk5/p35 for 48 h (*n* = 28) in comparison with control cells. Scaled current density-voltage relationships confirmed that co-expression of the Cdk5/p35 complex has a stimulatory effect on current density in these cells (Fig. 4D).

**Fig 5:**
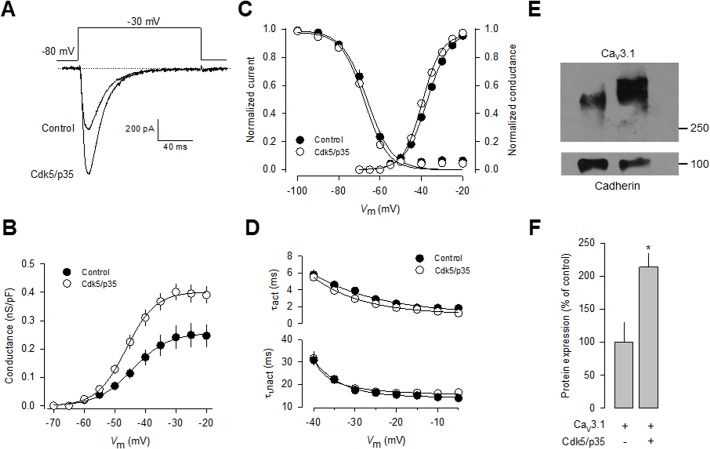
Regulation of recombinant CaV3.1 protein membrane expression by Cdk5. A) Representative macroscopic current traces recorded from HEK-293 cells transiently transfected with a plasmid encoding Ca_V_3.1 channels alone or in conjunction with plasmids encoding Cdk5 and p35. Currents were elicited by depolarizing steps from a *V*_h_ of −80|mV to-30|mV. Ba^2+^ (2 mM) was used as the charge carrier. B) Conductance-voltage (*G-V*) curves were calculated for each cell. An increase in conductance was observed in Cdk5/p35-coexpressing cells. C) Cdk5/p35 overexpression in HEK-293 cells stably expressing Ca_V_3.1 channels did not affect the voltage dependence of current activation and steady-state inactivation. D) Comparison of the time constant of current activation (τ_act_) and inactivation (τ_inact_) at different membrane voltages in untransfected (control) and Cdk5/p35-coexpressing cells. E) Representative cell surface protein extraction assay followed by Western blot using an anti-Ca_V_3.1 specific antibody. F) Densitometric quantification of three repetitions of the experiment shown in E. Asterisk denotes significant differences (P>0.05) between cells expressing the Ca_V_3.1 channels only, and cells transfected with the channels plus the Cdk5/p35 complex.

In contrast to the significant effect of Cdk5/p35 on current density and in conductance (Fig. 5A-B), the voltage dependence of channel activation and inactivation was not significantly altered (Fig. 5C). In addition, the time constants for current activation and inactivation were not also significantly modified (Fig. 5D). These data are consistent with the results from the recordings obtained in N1E-115 cells (Figs. 1C and 3E). With a view to gaining further insight into the mechanisms by which Cdk5 is affecting Ca_V_3.1 channel currents, we examined whether the surface expression of the Ca_V_3.1 channel protein was altered. To this end, we measured the expression of the channels in plasma membrane protein extracts. By using the membrane-bound adhesion molecule E-cadherin as a control, we found a significant increase in Ca_V_3.1 subunit in HEK-293 cells transiently co-transfected with Cdk5/p35 compared with the control (Fig. 5E-F).

**Fig 6:**
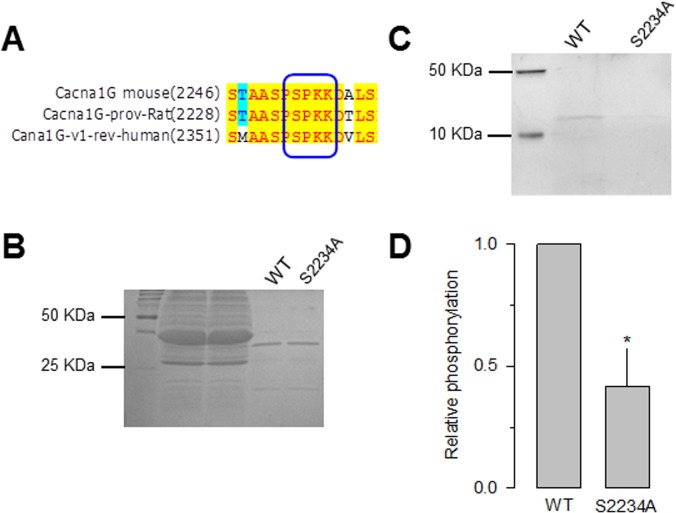
Phosphorylation of the CaV3.1 channel in vitro at Serine 2234. A) Amino acid sequence alignment of a small region within the Ca_V_3.1 channel C-terminus showing a consensus Cdk5 phosphorylation site conserved among three species. The box marks the sequence that matches the Cdk5 consensus site with the phosphorylatable serine at position 2234. Direct DNA sequencing of PCR products confirmed that the construct generated by site-directed mutagenesis of the Cdk5 consensus phosphorylation site contained the mutant sequence. B) For *in vitro* Cdk5-mediated phosphorylation assays GST-Ca_V_3.1 fusion wild-type (WT) and S2234A mutant proteins were purified on glutathione agarose beads, fractionated by SDS-PAGE and stained with Coomassie blue. C) Purified recombinant Ca_V_3.1 wild-type and S2234A constructs (amino acid residues 2154 to 2254) were subjected to phosphorylation by purified Cdk5/p25 complex *in vitro*. D) Comparison of the relative *in vitro* phosphorylation levels GST-Ca_V_3.1 fusion WT and S2234A mutant proteins (*n* = 3).

In parallel, we searched for the presence of the consensus sequence for Cdk5 phosphorylation [32] in the Ca_V_3.1 channel sequence using the database publicly available at the URL http://gps.biocuckoo.org/. We found several sites in Ca_V_3.1 as possible Cdk5 substrates. This analysis showed four sites T539, T541, S2232, S2234, with high scores (11.9, 11.2, 11.4 and 11.7, respectively). The first two sites were located in the I-II loop of the Ca_V_α1 subunit while the other two were in the C-terminal of the protein. In particular, serine 2234 was identified as the major site of Cdk5 phosphorylation (Fig. 6A) because it was conserved among species and the last amino acid in the consensus site corresponded to a lysine. The identity of this site as a possible Cdk5 substrate was further confirmed using a novel systematic computational search strategy for putative phosphorylation sites for Cdk5 in the mouse proteome developed previously by our research group [33]. This strategy uses a position specific scoring matrix (PSSM) based on a manually curated dataset of sites shown to be phosphorylated both *in vivo* and *in vitro* by Cdk5. Due to the highly stringent filtering criteria the use of this strategy significantly reduces the number of false positives [33]. PSSM analysis showed that the Serine residue at position 2361 in the mouse Cav3.1 sequence (S2234 in the rat sequence) has a very high score as putative site for Cdk5-mediated phosphorylation, and it is likely to be phosphorylated based on the results of phosphoproteomics studies of the mouse brain.

To determine whether this site is phosphorylated by Cdk5, we generated a point mutation in which serine 2234 was substituted with alanine by site-directed mutagenesis. Carboxyl-terminal GST fusion proteins containing the region of interest were then generated, expressed in BL21 cells and purified for analysis using an *in vitro* phosphorylation assay. Coomassie blue gel staining showed that the total proteins of the lysate applied in each lane of the gels were very similar (Fig. 6B). Interestingly, Pro-Q Diamond gel staining showed that Cdk5 phosphorylated the wild-type C-terminus Ca_V_3.1 GST fusion protein, whereas the S2234A mutant construct displayed low levels of phosphorylation (Fig. 6C-D).

**Fig 7:**
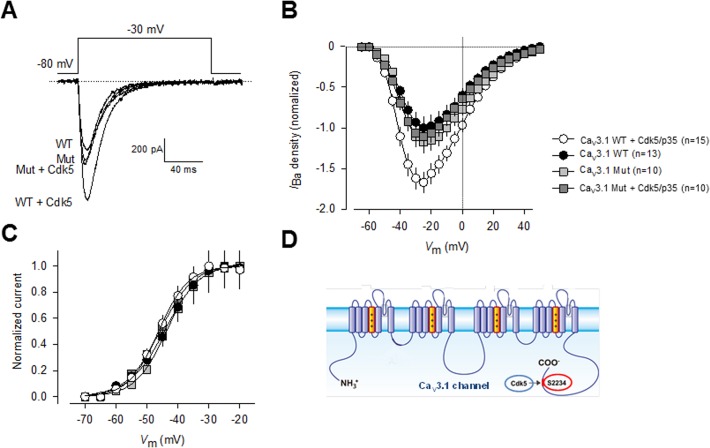
Mutation of Serine 2234 abolishes Cdk5/p35-mediated regulation of CaV3.1 channels. A) Representative macroscopic current traces recorded from HEK-293 cells transiently transfected with plasmid constructs encoding wild-type and S2234A mutant Ca_V_3.1 channels alone or in conjunction with plasmids encoding Cdk5 and p35. Currents were elicited by depolarizing steps from a *V*_h_ of −80 mV to −30 mV. Ba^2+^ (2 mM) was used as the charge carrier. B) Comparison of normalized current density-voltage relationships in wild-type and S2234A mutant Ca_V_3.1 channel transfected HEK-293 cells in the presence or absence of the Cdk5/p35 complex. C) Cdk5/p35 coexpression in HEK-293 cells expressing the wild-type and S2234A mutant channels did not affect the voltage dependence of current activation. The normalized *G-V* curves were fit by a single Boltzmann as described in Methods. Data analysis showed negligible differences in *V*_½_ or the slope factor (*k*). D) The Ca_V_3.1 T-type channels and the Cdk5/p35 complex may interact resulting in a Cdk5/p35-mediated increase in channel cell surface expression and current density, via a functional phosphorylation site at S2234 located within the C-terminus of the Ca_V_3.1_α1_ protein.

We next tested whether the S2234 might be a functional Cdk5 phosphorylation site, by performed site-directed mutagenesis and electrophysiological recordings. First, a construct encoding the full-length Ca_V_3.1 cDNA was transfected transiently into HEK-293 cells, and the resulting channels were studied using the whole-cell patch clamp technique. It is worth mentioning that the transfected channels expressed well in HEK-293 cells (Fig. 7A), had current densities similar to those obtained with the stably transfection, and produced typical current waveforms expected from T-type Ca_V_3.1 channels. As expected, current density was conspicuously larger in cells cotransfected with Cdk5/p35 at almost all voltages tested (Fig. 7B), but no differences in current kinetics were evident. Consistent with this, activation curves showed a significant ∼1.5-fold increase in conductance in the cells coexpressing Cdk5/p35, though scaled activation curves did not differ significantly in the absence and presence of Cdk5/p35 (Fig. 7C).

Based on the *in silico* analysis and the *in vitro* phosphorylation assay, we hypothesized that Ca_V_3.1 activity would be dependent on the phosphorylation state of Ser2234. Accordingly, Cdk5 would not be expected to increase currents carried by Ca_V_3.1 channels harboring the mutation. This was confirmed in whole cell patch clamp experiments performed in HEK-293 cells transiently transfected with the wild-type or the mutant Ca_V_3.1 channel cDNA alone or in conjunction of the Cdk5 cDNA. As shown in Fig. 7A and 7B, Ser2234 mutant Ca_V_3.1 channels were refractory to modulation by the Cdk5/p35 complex. Likewise, as observed in currents recorded through native T-type channels in N1E-115 cells and recombinant Ca_V_3.1 channels stably expressed in the HEK-293 cell line, the voltage dependence of activation of the currents through Ca_V_3.1 mutants did not differ significantly from that observed with the wild type channels, both in the absence and presence of Cdk5/p35 (Fig. 7B). No differences in voltage dependence or current kinetics were evident.

## Discussion

Diverse studies have shown that Cdk5 has multiple functions in the developing, adult, and aging brain, and that a change in its localization from the growth cones to the presynaptic terminals is an important mechanism underlying the diversity of functions Cdk5 plays in nerve cells [34],[35],[36]. However, little is known concerning the regulatory effects of this kinase on Ca^2+^ channels, essential regulators of neuronal activity.

Previous reports have shown that Cdk5 inhibitors favor neurotransmitter release by increasing the activity of P/Q-type channels. In this regard, initial work by Tomizawa and colleagues showed that Cdk5 may phosphorylate the II-III loop in the pore-forming (Ca_V_2.1α_1_) subunit, inhibiting the interaction of the channels with SNARE proteins such as SNAP-25 and synaptotagmin [12]. These results suggested that Cdk5 plays a key role in neurotransmitter release at the presynaptic terminals of the adult brain.

More recently, Su and colleagues showed that the N-type (Ca_V_2.2) channel, the other major presynaptic Ca^2+^ channel, is also a substrate of Cdk5. Phosphorylation of the pore-forming Ca_V_2.2α _1_ subunit by Cdk5 increased Ca^2+^ influx and facilitated neurotransmitter release by enhancing channel open probability (P_o_). These events seemed to be mediated by an interaction between Ca_V_2.2α_1_ and RIM1, which controls vesicle docking at the active zones. These results outlined a mechanism by which Cdk5 may regulate N-type channels and consequently might determine presynaptic function [37]. Likewise, these studies corroborated the pivotal role for Cdk5-mediated phosphorylation of Ca_V_ channels in regulating presynaptic function, and highlighted the close interaction between kinases and these channels in neurons.

Here, studying native T-type channels expressed in N1E-115 cells, as well as recombinant channels transiently and stably expressed in HEK-293 cells, we show that Ca_V_3.1 channels, which play a crucial role in determining neuronal excitability, are also regulated by Cdk5-mediated phosphorylation. The functional relevance of the Cdk5-mediated phosphorylation on Ca_V_3.1 channel activity was firstly assessed by whole-cell recordings in Cdk5 transfected N1E-115 cells. Remarkably, following coexpression of Cdk5 peak current amplitude and current density were significantly increased compared to control (GFP transfected) cells. Likewise, in a cell line stably expressing Ca_V_3.1, Cdk5 transfection also increased current density, providing independent support for the notion that the increase in native neuronal T-type current density observed in N1E-115 cells may be mediated by Cdk5-mediated phosphorylation. In addition, we conducted whole-cell recordings in HEK-293 cells from transiently transfected with either the wild-type or a phosphorylation mutant of Ca_V_3.1 channels, in which a Cdk5 phosphorylation site (S2234A) in the C-terminal region was abolished. In these experiments, we found that the phosphorylation mutant expressed a current density profile similar to that of wild-type Ca_V_3.1; however, cells in the presence of Cdk5 did not display an increase in current density.

Distinct mechanisms may underlie the increase in Ca_V_3.1 current density following Cdk5-mediated phosphorylation. Previous studies have shown a role for diverse post-translational modifications in the regulation of T-type channels and enhanced Ca^2+^ influx due to increased channel surface expression [38],[39],[40]. Indeed, this could be the most plausible explanation for our results, considering particularly that there were no significant differences in activation kinetics or voltage dependence of activation and inactivation between currents through Ca_V_3.1 channels in the presence or absence of Cdk5. In this regard, the role of Cdk5 in channel forward trafficking or in endocytosis is an interesting issue that remains unexplored.

In addition, it is worth mentioning that Cdk5-mediated phosphorylation increases P_o_ of Ca_V_2.2 (N-type) channels, and that this regulation is abolished in a mutant channel in which all Cdk5 phosphorylation sites in the Ca_V_2.2α_1_ protein were eliminated [37]. Furthermore, it has been shown that protein kinase C (PKC) can modulate Cav3 channels activity but pharmacological and fluorescence studies revealed that the surface density of Cav3.1 T-type channels was not significantly changed by activation of PKC, suggesting that Ca_V_3.1 channel P_o_ might be affected by phosphorylation [41]. It would be of great interest, therefore, to investigate whether phosphorylation of the Ca_V_3.1 channel by Cdk5 also involves changes in channel P_o_.

The physiological relevance of phosphorylation of native T-type channels and their contribution to the function of the cells expressing these channels has not been totally understood. It is well recognized that Ca_V_ channels modulate the function of different neuronal types by influencing synaptic transmission and neuronal excitability. While HVA Ca_V_ channels participate in fast synaptic transmission, T-type channels have a unique function in neuronal excitability [2]. Neuronal T-type channels have been shown to promote burst firing and low-amplitude intrinsic neuronal oscillations, as well as Ca^2+^ entry and amplification of dendritic synaptic signals. Interestingly, evidence obtained in the last few years show that protein kinase activity may impact T-type channel function in different manners [42]. In this scenario, Cdk5 can profoundly affect cellular excitability by increasing the amplitude of the T-type currents.

Last, as mentioned earlier, Cdk5 is a serine/threonine kinase that is activated upon association with its activators p35 and p39 (non-cyclin proteins). It is predominantly neuronal, though Cdk5 can be active also in several non-neuronal tissues. Cdk5 plays a pivotal role in the CNS development, and Cdk5 dysregulation has been implicated in different pathologies including Alzheimer's and Parkinson's disease [43]. Interestingly, it has been reported that nerve-growth factor (NGF) induces strong, sustained expression of p35 through activation of the ERK pathway [44]. The induced kinase activity of Cdk5 may be required for NGF-induced neurite outgrowth. These results suggest the possibility of Cdk5 activation directly via the NGF receptor expressed heterologously in conjunction with recombinant Ca_V_3.1 channels while performing electrophysiological recordings. This is a very interesting topic for future experiments.

In summary, here we demonstrated a previously uncharacterized interaction between Ca_V_3.1 and the Cdk5/p35 complex that results in a Cdk5/p35-mediated increase in Ca_V_3.1 current density, and identified a major phosphorylation site at serine 2234 within the C-terminus of the Ca_V_3.1α_1_ pore forming subunit (Fig. 7D). These findings provide a framework to examine how Ca_V_3.1 channels are regulated in the context of Cdk5 physiological activity.
